# On the genus *Flexicrurum* Tong & Li, 2007 (Araneae, Psilodercidae) from Hainan Island, China

**DOI:** 10.3897/zookeys.855.34383

**Published:** 2019-06-13

**Authors:** Wan-Jin Chang, Fengyuan Li, Shuqiang Li

**Affiliations:** 1 Institute of Zoology, Chinese Academy of Sciences, Beijing 100101, China Institute of Zoology, Chinese Academy of Sciences Beijing China

**Keywords:** Endemic, Ochyroceratidae, Southeast Asia, spider, taxonomy, tropical

## Abstract

Three new species of the genus *Flexicrurum* Tong & Li, 2007, from Hainan Island, China are described: *F.wuzhishanense***sp. nov.** (♂♀), *F.yangjiao***sp. nov.** (♂♀), and *F.qishi***sp. nov.** (♂♀). A key to males of species of *Flexicrurum* is provided. Additionally, the female of *F.minutum* Tong & Li, 2007, is described for the first time. To date, the genus is endemic to Hainan Island, China. Types are deposited in the Institute of Zoology, Chinese Academy of Sciences (IZCAS) in Beijing.

## Introduction

The spider family Psilodercidae Machado, 1951, was only recently elevated to family rank from a subfamily of Ochyroceratidae Fage, 1912. This taxonomic rearrangement is based on the presence of book-lungs, the position of tracheal stigma, the number of promarginal cheliceral teeth, the shape of the labium, and the point of attachment of the bulbus (Wunderlich 2004, 2008).

Psilodercidae currently contains 11 genera and 120 species (World Spider Catalog 2019; Li and Quan 2017). Psilodercids are restricted to tropical Asia and are diverse within China. Thirteen species from six genera have been reported in China: *Althepuschengmenensis* Li & Li, 2018, *A.christae* Wang & Li, 2013, *A.menglaensis* Li & Li, 2018, *A.qingyuani* Li & Li, 2018, *A.xuae* Li & Li, 2018, *Leclerceraundulata*Wang & Li, 2013, and *Psilodercesincomptus* Wang & Li, 2013, from Yunnan Province; *Flexicrurumflexicrurum* Tong & Li, 2007, *F.longispina* Tong & Li, 2007, *F.minutum* Tong & Li, 2007, and *Qiongocerahongjunensis* Li & Li, 2017, from Hainan Island; and *Sinodercesexilis* Wang & Li, 2013, and *S.nawanensis* Li & Li, 2017, from Guangxi Zhuang Autonomous Region. All of them are locally endemic.

Prior to this study, only three species from the genus *Flexicrurum* Tong & Li, 2007, have been described (Tong and Li 2007): *F.flexicrurum*, *F.longispina*, and *F.minutum*. These species are confined to Hainan Island, China, and have been the only representatives of the genus (World Spider Catalog 2019).

While studying new material collected in Hainan Island, we recognized the matched pairs of three new species of *Flexicrurum*, and a hitherto unknown female of *F.minutum*, one of the three species described by Tong and Li (2007). This paper describes all of these new discoveries by providing images of their genital organs and close-up photos of their chelicerae.

## Materials and methods

Types are deposited in the Institute of Zoology, Chinese Academy of Sciences (**IZCAS**) in Beijing. All specimens collected were observed and preserved in 95% ethanol. The specimens were measured and examined under a Leica M205 C stereomicroscope, and further morphological details were observed with an Olympus BX41 compound microscope. The left male palp was detached for closer examination. Carapace measurements include the clypeus. The internal genitalia and male bulb were dissected and immersed in lactic acid. An Olympus C7070 wide zoom digital camera (7.1 megapixels) mounted on an Olympus SZX12 stereomicroscope was used to take photos. Photos were stacked with Helicon Focus 6.7.1 to generate images with extended depth of field. The images were post-processed with Adobe Photoshop. Leg measurements are shown as total length (femur, patella, tibia, metatarsus, and tarsus). Leg segments were measured from the retrolateral side. All measurements are given in millimetres (mm). Terminology follows that of Li et al. (2014), Tong and Li (2007) and Deeleman-Reinhold (1995). Coordinates of collecting locations were recorded in Microsoft Excel and imported into ArcGIS 10.2 to generate a map which was subsequently exported to Adobe Photoshop CC 2014 for further editing. The following abbreviations are used in text: **ALE** anterior lateral eye, **ME** median eye, **PLE** posterior lateral eye.

## Taxonomy

### Family Psilodercidae Machado, 1951

#### Genus Flexicrurum

Taxon classificationAnimaliaAraneaeOchyroceratidae

Tong & Li, 2007

##### Type species.

*Flexicrurumflexicrurum* Tong & Li, 2007 from China, Hainan Island, Wuzhishan, Wuzhishan City, 16.IV.2005 (IZCAS).

##### Emended diagnosis.

*Flexicrurum* Tong & Li, 2007, resembles *Althepus* Thorell, 1898, and *Leclercera* Deeleman-Reinhold, 1995. However, *Flexicrurum* can be differentiated by the combination of the following characteristics: 1) the tibia of the male palp is strongly rotated inward (vs absence of rotated male palpal tibia in *Althepus* and *Leclercera*); 2) presence of a slender bulbal apophysis (vs absence of slender bulbal apophysis in *Althepus* and *Leclercera*); 3) cymbium with a strong lateral protrusion (vs cymbium with slightly tilted protrusion in *Althepus* and *Leclercera*); 4) cymbium bearing a one- or two-bulge posterolateral cymbial apophysis (vs cymbium with lateral lanceolate or hook-shaped, spine like apophysis in *Althepus*; cymbium with retrolateral apophysis in *Leclercera*); 5) absence of promarginal cheliceral teeth (vs 1–2 promarginal cheliceral teeth in *Althepus* and *Leclercera*); 6) embolus distinctly short (vs embolus long and slender in *Althepus* and *Leclercera*); 7) internal endogyne with the presence of a distinct vertical duct medially bearing different structures of spermathecae (vs absence of the vertical duct in *Althepus* and *Leclercera*).

##### Composition.

*Flexicrurumflexicrurum* Tong & Li, 2007, *F.longispina* Tong & Li, 2007, *F.minutum* Tong & Li, 2007, *F.qishi* Li & Li, sp. nov., *F.wuzhishanense* Li & Li, sp. nov., and *F.yangjiao* Li & Li, sp. nov.

##### Distribution.

Hainan Island, China.

#### Key to species of *Flexicrurum* (males only)

**Table:** 

1	Cymbium with posterolateral apophysis with a divided tip, i.e., with two bulges or protuberances	**2**
–	Cymbium with posterolateral apophysis with a single tip or bulge	**3**
2	Conductor simple, protruded; longer bulbal apophysis; bulb with scattered black spots	***F.wuzhishanense* sp. nov.**
–	Conductor comprising two parts that spiral inwards, resembling a broken ring; shorter bulbal apophysis; bulb without scattered black spots	***F.qishi* sp. nov.**
3	Laminar apophysis and tip of cymbial protrusion parallel to one another	**4**
–	Laminar apophysis and tip of cymbial protrusion not parallel to one another	***F.yangjiao* sp. nov.**
4	Absence of long spine on dorsolateral surface of bulb	**5**
–	Presence of a long spine on dorsolateral surface of bulb	*** F. longispina ***
5	Longer bulbal apophysis (exceeds length of entire bulb)	*** F. flexicrurum ***
–	Shorter bulbal apophysis (does not exceed length of entire bulb)	*** F. minutum ***

##### Flexicrurum wuzhishanense

Taxon classificationAnimaliaAraneaeOchyroceratidae

Li & Li sp. nov.

http://zoobank.org/7295226A-D99A-458F-BAEB-1E536F77452D

Figs 1 , 2 , 8A , 9

###### Types.

**Holotype**: ♂ (IZCAS), China, Hainan Island, Wuzhishan City, Shuiman Village, Wuzhishan, 18°54.1944'N, 109°40.9266'E, 723 m, 31.III.2012, Chen Z. leg.; **Paratype**: 1♀ (IZCAS), same data as holotype.

###### Etymology.

The species name is an adjective referring to the type locality; the Chinese pinyin “wǔ zhǐ” means five fingers, and “shān” means mountain. The name is a graphic interpretation of contour of the mountain ranges with a striking resemblance to five fingers.

###### Diagnosis.

**Figure 1.**
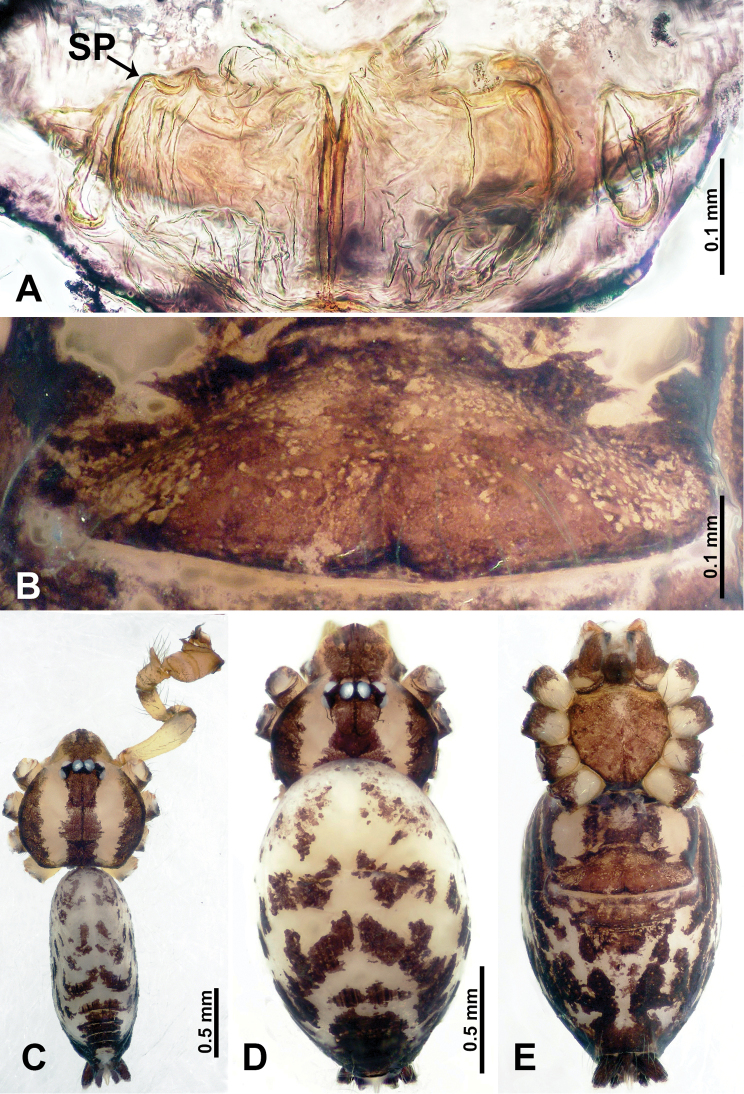
*Flexicrurumwuzhishanense* Li & Li, sp. nov., male holotype and female paratype **A** internal genitalia, dorsal view **B** female epigastric area, ventral view **C** male habitus, dorsal view **D** female habitus, dorsal view **E** female habitus, ventral view. Abbreviation: SP = spermathecae. **D** and **E** share the scale bar.

**Figure 2.**
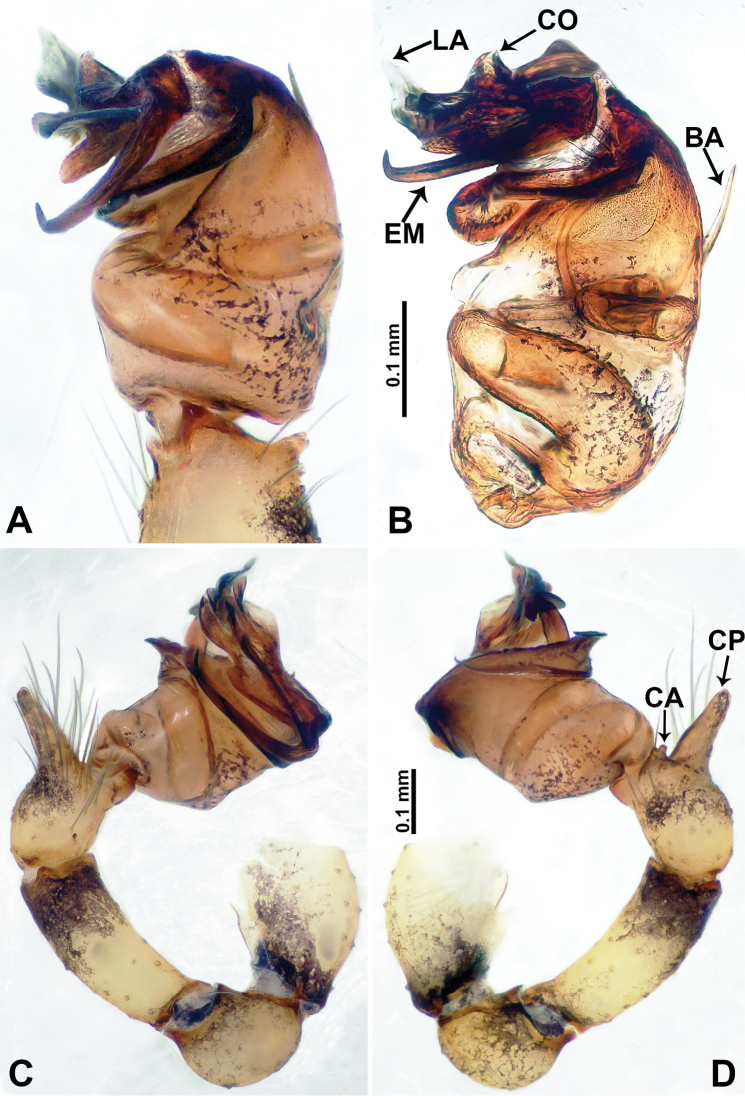
*Flexicrurumwuzhishanense* Li & Li, sp. nov., male holotype **A** palp, ventral view **B** palpal bulb, ventral view **C** palp, prolateral view **D** palp, retrolateral view. Abbreviations: BA = bulbal apophysis, CA = cymbial apophysis, CO = conductor, CP = cymbial protrusion, EM = embolus, LA = laminal apophysis. **A** and **B** share the scale bar as well as **C** and **D**.

*Flexicrurumwuzhishanense* sp. nov. strongly resembles *F.qishi* sp. nov. Males of *F.wuzhishanense* sp. nov. can be distinguished by a longer bulbal apophysis (Fig. 2D) (vs shorter bulbal apophysis in *F.qishi* sp. nov.), the bulbal apophysis which is further from embolus (Fig. 2B) (vs bulbal apophysis and embolus nearer each other (Fig. 6B), simple protruded conductor comprises only a single part (Fig. 2B) (vs a rather slender conductor comprising two parts, resembling a broken ring in *F.qishi* sp. nov. (Fig. 6A, B)), the bulb with scattered black spots (Fig. 2A) (vs absence of scattered black spots on bulb of *F.qishi* sp. nov. (Fig. 6A)); females can be distinguished by a rather broad dome-shaped epigastric area (Fig. 1B) (vs a rather plump, triangular epigastric area in *F.qishi* sp. nov. (Fig. 5B)), spermathecae can be distinguished by a pair of lobed ducts laterally connected with bow-tie-shaped spermathecae (Fig. 1A) (vs a pair of spermathecae resembling the structure of a human uterus in *F.qishi* sp. nov. (Fig. 5A)).

###### Description.

**Male** (Holotype). Total length 2.46; carapace 0.96 long, 0.94 wide; abdomen 1.50 long, 0.64 wide. Carapace round and brown, with three longitudinal dark brown bands, the middle band is 2 times wider than the lateral bands (Fig. 1C). Fovea shallow and dark brown. Anterior part of thoracic region distinctly elevated. Eye sizes and interdistances: ALE 0.09, ME 0.08, PLE 0.06; ALE–ALE 0.30, ME–ME 0.16, PLE–PLE 0.32, ALE–ME 0.14, PLE–ME 0.18, ALE–PLE 0.12. Chelicerae brown. Cheliceral promargin with lamina of three triangular extensions and no teeth, retromargin with two small teeth (Fig. 8A). Clypeus slanting 0.3 high, with dark brown trident and two pale areas laterally. Endites dark brown. Labium slanting and dark brown. Sternum circular with brown complex pattern delimiting a medial small pale spot anteriorly. Abdomen elongated, anterior of ventrum with pair of circular pale areas and dome-shaped epigastric area, posterior with random irregular dark brown spots. Legs uniformly brown; measurements: missing (detached from specimens, sequence of legs cannot be differentiated). Palp (Fig. 2A–D): femur slender swollen at the base, patella swollen and angled ventrally (Figs 1C, 2C), tibia more slender than femur, distally darker, cymbium pale, darker distally, with strong lateral protrusion darker distally, bearing posterolateral cymbial apophysis with two bulges (Fig. 2D); bulb brown and pyriform, with scattered dark spots, bearing a pointed laminar apophysis, a protruded conductor and embolus distally; embolus slender, hook-shaped, adjacent to laminar apophysis and conductor (Fig. 2B).

**Female** (Paratype). General features and coloration similar to male (Fig. 1D–E). Measurements: total length 2.12; carapace 0.75 long, 0.78 wide; abdomen 1.37 long, 0.93 wide. Eye sizes and interdistances: ALE 0.09, ME 0.06, PLE 0.08; ALE–ALE 0.30, ME–ME 0.14, PLE–PLE 0.32, ALE–ME 0.14, PLE–ME 0.18, ALE–PLE 0.15. Clypeus 0.35 high. Leg measurements: I missing, II missing, III missing, IV 7.38 (1.92, 0.31, 2.03, 2.03, 1.09). Internal genitalia: a pair of lobe-shaped ducts connected with a bow-tie-shaped spermathecae, divided by a distinct pair of vertical ducts with a pair of club-shaped ducts anteriorly (Fig. 1A).

###### Distribution.

Known only from the type locality (Fig. 9).

##### Flexicrurum yangjiao

Taxon classificationAnimaliaAraneaeOchyroceratidae

Li & Li sp. nov.

http://zoobank.org/1FDF0627-CE14-4FCB-BA69-2B88FC6EB417

Figs 3 , 4 , 8B , 9

###### Types.

**Holotype**: ♂ (IZCAS), China, Hainan Island, Changjiang City, Bawangling, Yajia Conference Centre, 19°5.1042'N, 109°7.4343'E, 433 m, 10.IV.2012, Chen Z. leg.; **Paratypes**: 1♂1♀ (IZCAS), same data as holotype.

###### Etymology.

The species name is a noun in apposition derived from the word for “goat horn” in Chinese pinyin “yángjiǎo”. It refers to the shape of the conductor which curves strongly inwards, like a goat horn.

###### Diagnosis.

*Flexicrurumyangjiao* sp. nov. can be distinguished from *F.wuzhishanense* sp. nov. and *F.qishi* sp. nov. by a posterolateral cymbial apophysis with a single tip or bulge (Fig. 4D) (vs a posterolateral cymbial apophysis with two bulges or protuberances (i.e., a divided tip) in *F.wuzhishanense* sp. nov. and *F.qishi* sp. nov.), a pointed embolic end (vs hook-liked embolic end in *F.wuzhishanense* sp. nov. and *F.qishi* sp. nov.), a shorter laminar apophysis (vs longer in *F.wuzhishanense* sp. nov. and *F.qishi* sp. nov.), and the position of the entire bulb is opposite that of the other two species—the laminar apophysis is parallel to the tip of cymbial protrusion (vs laminar apophysis and tip of cymbial protrusion not parallel in *F.wuzhishanense* sp. nov. and *F.qishi* sp. nov.).

**Figure 3.**
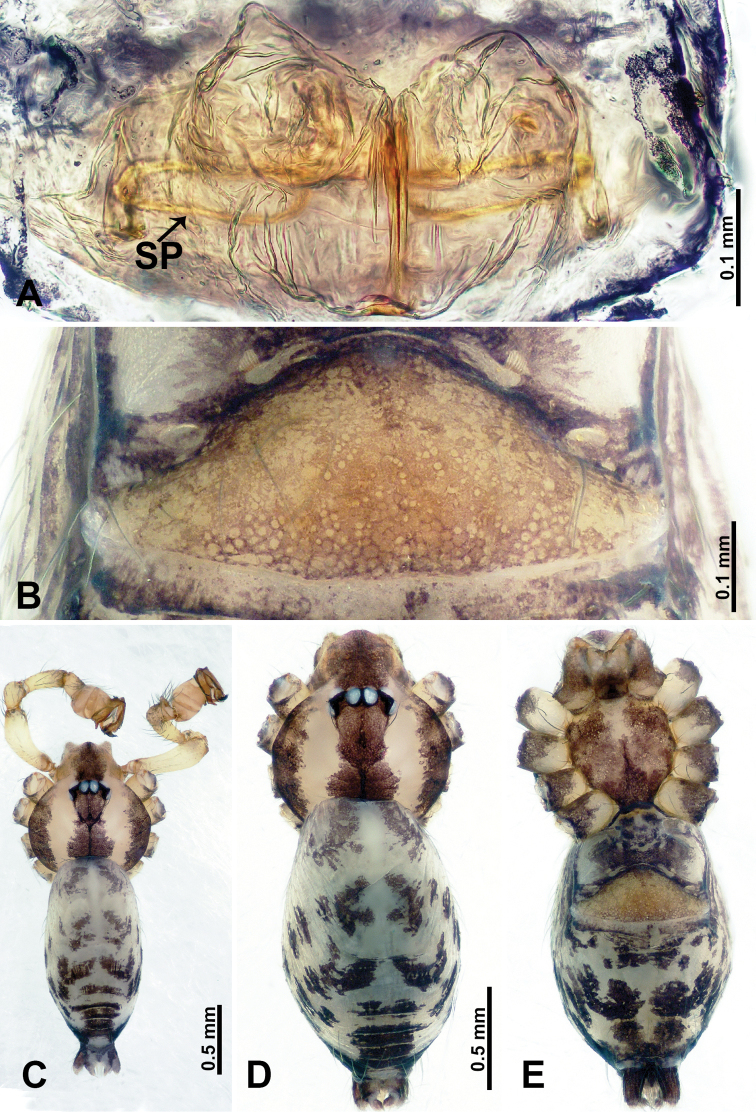
*Flexicrurumyangjiao* Li & Li, sp. nov., male holotype and female paratype **A** internal genitalia, dorsal view **B** female epigastric area, ventral view **C** male habitus, dorsal view **D** female habitus, dorsal view **E** female habitus, ventral view. Abbreviation: SP = spermathecae. **D** and **E** share the scale bar.

**Figure 4.**
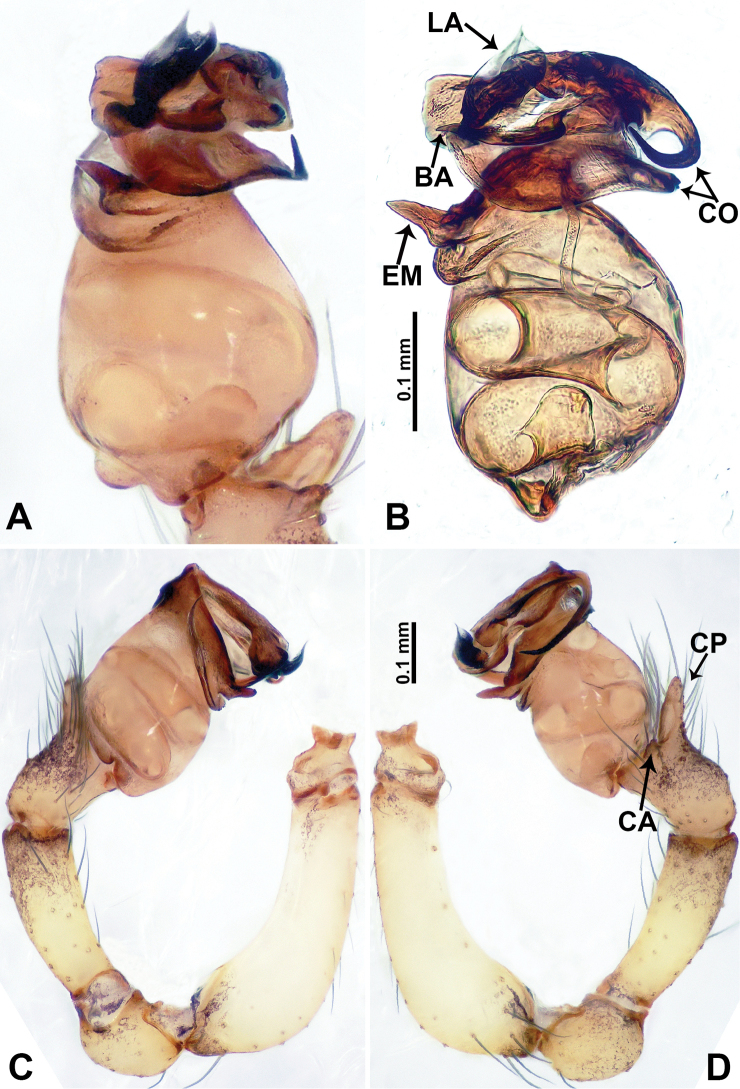
*Flexicrurumyangjiao* Li & Li, sp. nov., male holotype **A** palp, ventral view **B** palpal bulb, ventral view **C** palp, prolateral view **D** palp, retrolateral view. Abbreviations: BA = bulbal apophysis, CA = cymbial apophysis, CO = conductor, CP = cymbial protrusion, EM = embolus, LA = laminal apophysis. **A** and **B** share the scale bar as well as **C** and **D**.

###### Description.

**Male** (Holotype). Total length 2.08; carapace 0.80 long, 0.96 wide; abdomen 1.28 long, 0.64 wide. Carapace round and brown, with three longitudinal dark brown bands, the middle band 2 times wider than the lateral bands (Fig. 3C). Fovea shallow and dark brown. Anterior part of thoracic region distinctly elevated. Eye sizes and interdistances: ALE 0.08, ME 0.08, PLE 0.09; ALE–ALE 0.30, ME–ME 0.13, PLE–PLE 0.32, ALE–ME 0.16, PLE–ME 0.19, ALE–PLE 0.16. Chelicerae brown. Cheliceral promargin with lamina of three triangular extensions and no teeth, retromargin with two small teeth (Fig. 8B). Clypeus slanting 0.20 high, medially dark brown and two pale areas laterally. Endites dark brown. Labium slanting and dark brown. Sternum circular with brown complex pattern delimiting a 1/3 medial pale spot anteriorly. Abdomen elongated, dorsum anterior 2/3 with random dark brown spots, posterior 1/3 with horizontal dark brown striated pattern medially, ventrum anterior half with pair of lobed pale areas and a dome-shaped epigastric area, posterior half with random, irregular dark brown spots. Legs uniformly brown; measurements: I 9.50 (2.56, 0.32, 2.88, 2.65, 1.09), II 6.64 (2.00, 0.25, 1.75, 1.71, 0.93), III 5.05 (1.20, 0.23, 1.37, 1.50, 0.75), IV missing. Palp (Fig. 4A–D): femur slender, swollen at the base, patella swollen and angled ventrally (Figs 3C, 4C), tibia swollen and dark distally, cymbium pale, darker distally, with strong lateral protrusion darker distally, bearing a posterolateral cymbial apophysis with a single bulge (Fig. 4D); bulb pale brown and pyriform, bearing a pointed laminar apophysis, a slender bulbal apophysis; conductor strongly spiralled forming a ring resembling a goat horn; embolus short and pointed, located below all other structures, further away from conductor (Fig. 4B).

**Female** (Paratype). General features and coloration similar to male (Fig. 3D–E). Measurements: total length 2.18; carapace 0.80 long, 0.80 wide; abdomen 1.38 long, 0.80 wide. Eye sizes and interdistances: ALE 0.08, ME 0.08, PLE 0.05; ALE–ALE 0.32, ME–ME 0.16, PLE–PLE 0.33, ALE–ME 0.13, PLE–ME 0.19, ALE–PLE 0.13. Clypeus 0.35 high. Leg measurements: I 7.34 (2.00, 0.25, 2.25, 1.75, 1.09), II missing, III 5.10 (1.28, 0.25, 1.75, 1.20, 0.62), IV 6.82 (1.71, 0.31, 2.00, 2.00, 0.80). Internal genitalia: a pair of ovoid ring-shaped spermathecae connected to bow-tie-shaped ducts divided by a pair of distinct vertical ducts, bearing a pair of droplet-shaped ducts laterally (Fig. 3A).

###### Distribution.

Known only from the type locality (Fig. 9).

##### Flexicrurum qishi

Taxon classificationAnimaliaAraneaeOchyroceratidae

Li & Li sp. nov.

http://zoobank.org/15FA5BD3-172D-4A38-8456-B21BB623D53F

Figs 5 , 6 , 8C , 9

###### Types.

**Holotype**: ♂ (IZCAS), China, Hainan Island, Tunchang County, Datong Village, Mountain Wolong, 19°27.5450'N, 110°7.3150'E, 248 m, 06.VII.2014, Li F. and Wang X. leg.; **Paratype**: 1♀ (IZCAS), same data as holotype.

###### Etymology.

The species name is a noun in apposition derived from the Chinese pinyin “qíshì” (knight) and refers to the ventral view of the bulb which resembles a piece in international chess game representing a knight (Fig. 6A).

###### Diagnosis.

The species is similar to *Flexicrurumwuzhishanense* sp. nov. Diagnostic features are discussed under *F.wuzhishanense* sp. nov.

###### Description.

**Figure 5.**
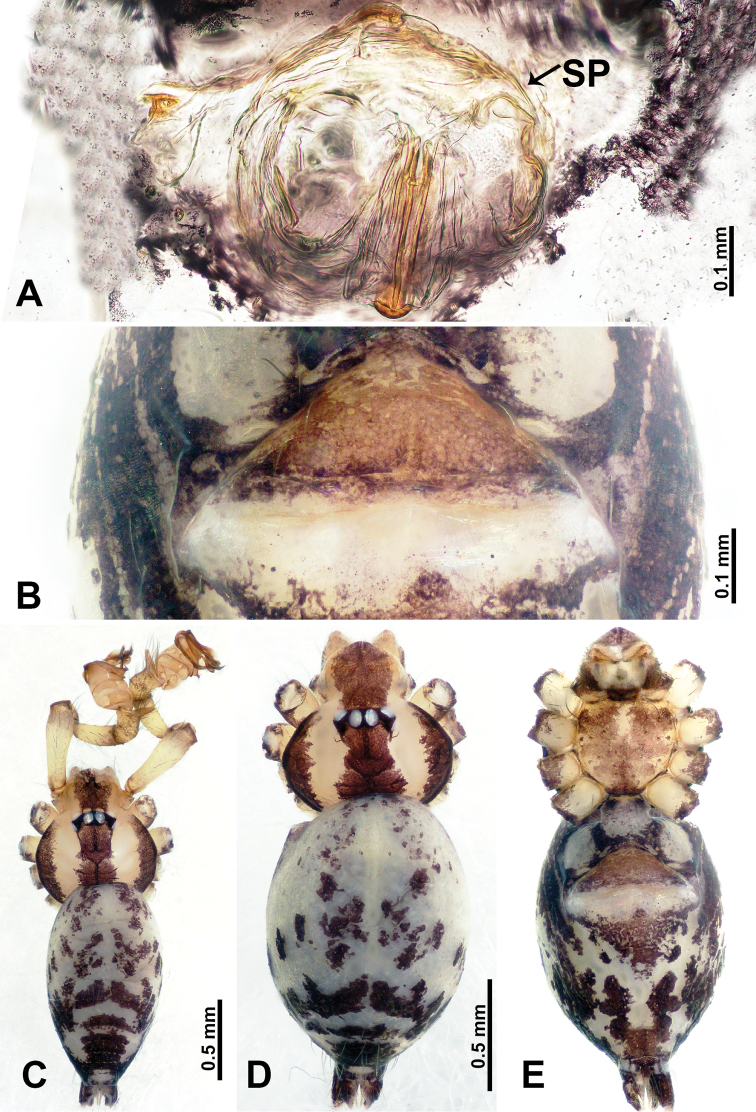
*Flexicrurumqishi* Li & Li, sp. nov., male holotype and female paratype **A** internal genitalia, dorsal view **B** female epigastric area, ventral view **C** male habitus, dorsal view **D** female habitus, dorsal view **E** female habitus, ventral view. Abbreviation: SP = spermathecae. **D** and **E** share the scale bar.

**Figure 6.**
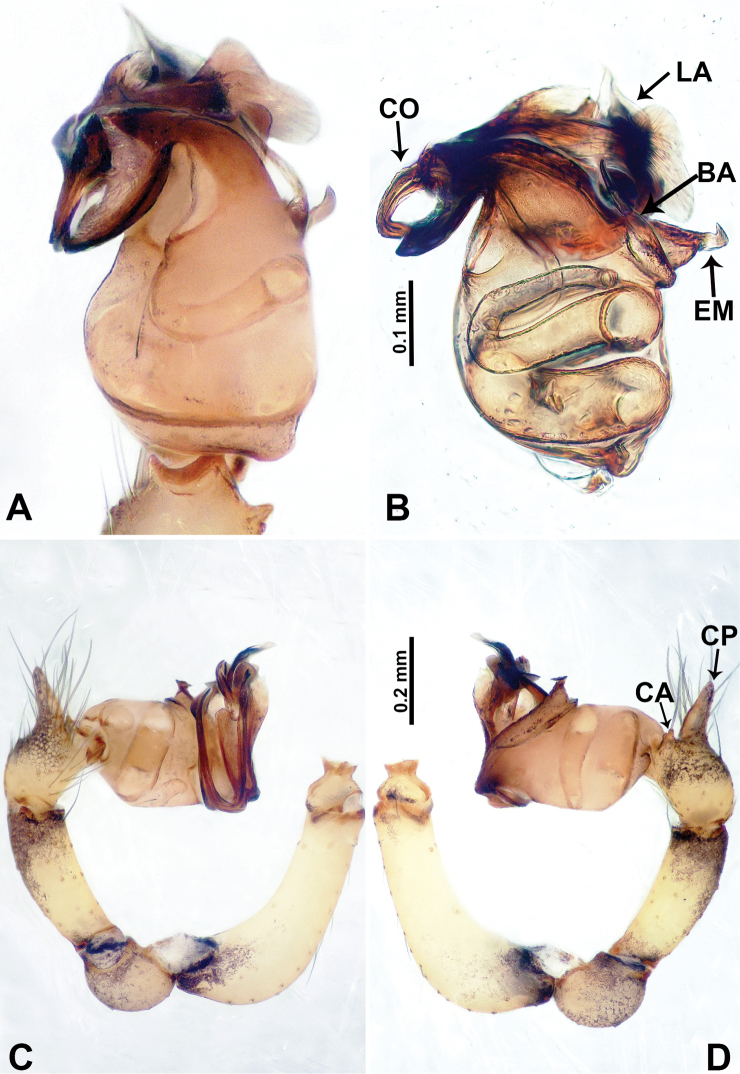
*Flexicrurumqishi* Li & Li, sp. nov., male holotype **A** palp, ventral view **B** palpal bulb, ventral view **C** palp, prolateral view **D** palp, retrolateral view. Abbreviations: BA = bulbal apophysis, CA = cymbial apophysis, CO = conductor, CP = cymbial protrusion, EM = embolus, LA = laminal apophysis. **A** and **B** share the scale bar as well as **C** and **D**.

**Male** (Holotype). Total length 2.45; carapace 1.25 long, 0.75 wide; abdomen 1.20 long, 0.68 wide. Carapace round and brown, with three longitudinal dark brown bands, the middle band is 2 times wider than the lateral bands (Fig. 5C). Fovea shallow and dark brown. Anterior part of thoracic region distinctly elevated. Eye sizes and interdistances: ALE 0.08, ME 0.06, PLE 0.05; ALE–ALE 0.28, ME–ME 0.15, PLE–PLE 0.30, ALE–ME 0.16, PLE–ME 0.18, ALE–PLE 0.13. Chelicerae brown. Cheliceral promargin with lamina of three triangular extensions and no teeth, retromargin with two small teeth (Fig. 8C). Clypeus slanting 0.20 high, medially dark brown and two pale areas laterally. Endites dark brown. Labium slanting and dark brown. Sternum circular with brown complex pattern delimiting a 1/3 medial small pale spot anteriorly and bottom margin. Abdomen elongated, dorsum anterior 2/3 with random dark brown spots, posterior 1/3 with horizontal dark brown striated pattern medially, ventrum anterior half with pair of circular pale areas and ovoid epigastric area, posterior half with random irregular dark brown spots. Legs uniformly brown; measurements: missing (detached from specimens, sequence of legs cannot be differentiated). Palp (Fig. 6A–D): femur slender, swollen at the base, patella swollen and angled ventrally (Figs 5C, 6C), tibia swollen and darker distally, cymbium pale, darker distally, with strong lateral protrusion darker distally, bearing posterolateral cymbial apophysis with two bulges (Fig. 6D); bulb pale brown and pyriform, bearing a pointed laminar apophysis, a slender bulbal apophysis adjacent to embolus which is located further away from conductor; conductor comprising two parts (two slender conductors circling inwards) resembling a broken ring; embolus hook-shaped, curving distally (Fig. 6B).

**Female** (Paratype). General features and coloration similar to male (Fig. 5D–E). Measurements: total length 2.15; carapace 0.75 long, 0.78 wide; abdomen 1.40 long, 0.93 wide. Eye sizes and interdistances: ALE 0.09, ME 0.06, PLE 0.05; ALE–ALE 0.29, ME–ME 0.13, PLE–PLE 0.31, ALE–ME 0.16, PLE–ME 0.18, ALE–PLE 0.14. Clypeus 0.23 high. Leg measurements: I missing, II missing, III missing, IV 6.65 (1.80, 0.25, 1.80, 1.87, 0.93). Internal genitalia: a pair of spermathecae resembling the structure of human uterus (distinct pair of lobe-shaped ducts hanging directed posteriorly, medially with a pair of vertical ducts bearing a curvy, flat duct posteriorly) (Fig. 5A).

###### Distribution.

Known only from the type locality (Fig. 9).

##### Flexicrurum minutum

Taxon classificationAnimaliaAraneaeOchyroceratidae

Tong & Li, 2007

Figs 7 , 9

Flexicrurum minutum Tong and Li, 2007: 65, figs 1I–L, 4A–E; Tong, 2013: 20, figs 16I–L, 35A–E

###### Type examined.

**Holotype**: ♂ (IZCAS), China, Hainan Island, Changjiang County, Bawangling National Natural Reserve, 22.III.2005.

###### Other material.

♀ (IZCAS), China, Hainan Island, Dongfang City, Donghe Town, Nanlang Village, foot of Mountain E-Xian, 19°0.3800'N, 109°5.0300'E, 214 m, 16.XII.2014, Zhao Q. and Shao L. leg.

###### Description.

**Figure 7.**
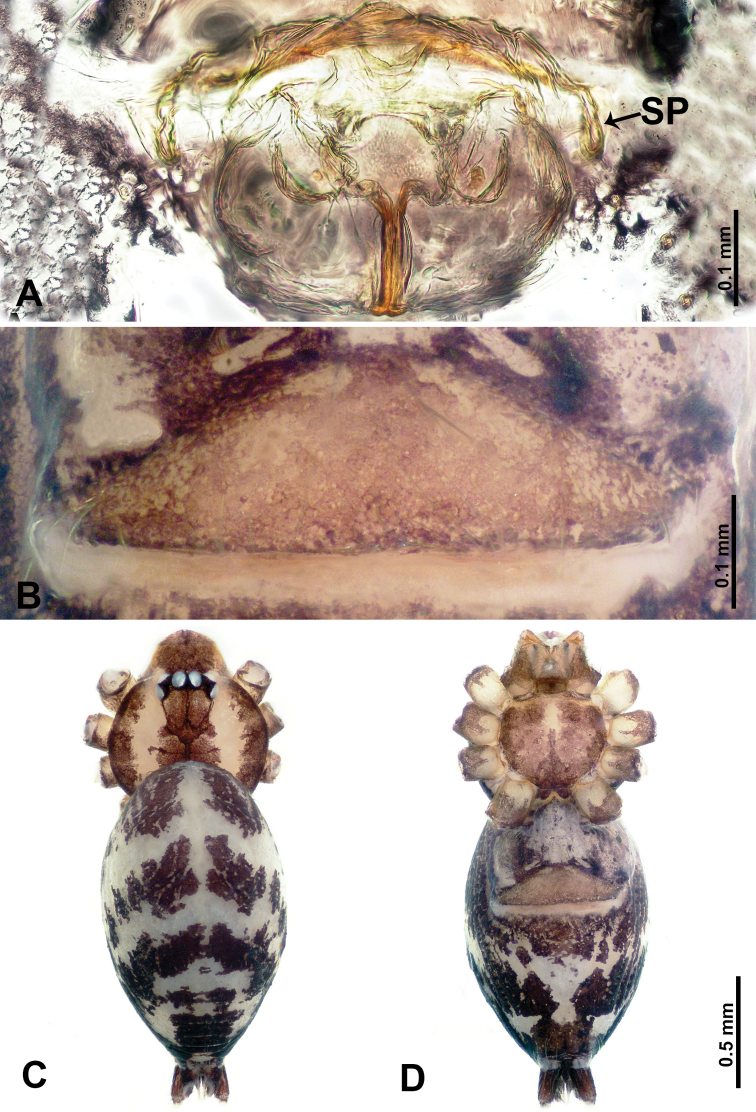
*Flexicrurumminutum* Tong & Li, 2007, female **A** internal genitalia, dorsal view **B** female epigastric area, ventral view **C** female habitus, dorsal view **D** female habitus, ventral view. Abbreviation: SP = spermathecae. **C** and **D** share the scale bar.

**Female**. Total length 2.34; carapace 0.78 long, 0.75 wide; abdomen 1.56 long, 0.87 wide. Carapace round and brown, with three longitudinal dark brown bands, the middle band is 2 times wider than the lateral bands (Fig. 7C). Fovea shallow and dark brown. Anterior part of thoracic region distinctly elevated. Eye sizes and interdistances: ALE 0.08, ME 0.06, PLE 0.05; ALE–ALE 0.31, ME–ME 0.15, PLE–PLE 0.33, ALE–ME 0.14, PLE–ME 0.18, ALE–PLE 0.15. Chelicerae brown. Cheliceral promargin with lamina of three triangular extensions and no teeth, retromargin with two small teeth (Tong and Li 2007). Clypeus slanting 0.24 high, dark brown. Endites dark brown. Labium slanting and dark brown. Sternum circular with brown complex pattern delimiting a medial ovoid pale spot anteriorly. Abdomen elongated, dorsum anterior 2/3 with dark brown striated spacing delimiting a pale area medially, posterior 1/3 with horizontal dark brown striated pattern medially, ventrum with brown pattern at margin and anterior half medially pale with an ovoid epigastric area, posterior half with random irregular dark brown pattern (Fig. 7D). Legs uniformly brown; measurements: I 7.85 (2.24, 0.25, 2.40, 1.87, 1.09), II 5.76 (1.60, 0.25, 1.60, 1.56, 0.75), III 5.02 (1.75, 0.20, 1.20, 1.25, 0.62), IV 7.11 (1.87, 0.25, 2.12, 2.00, 0.87). Internal genitalia: a pair of umbrella-shaped spermathecae (a pair of vertical ducts posteriorly connected with pair of ovoid vesicle and covered by a dome anteriorly) (Fig. 7A).

**Figure 8.**
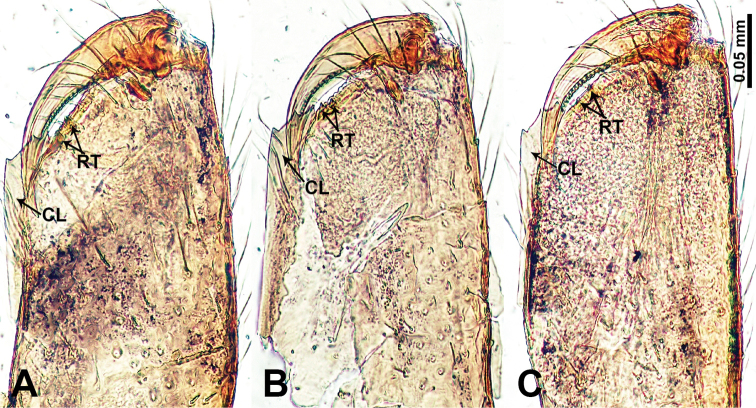
Cheliceral retromargin **A***Flexicrurumwuzhishanense* Li & Li, sp. nov. **B***F.yangjiao* Li & Li, sp. nov. **C***F.qishi* Li & Li, sp. nov. Abbreviations: CL = cheliceral laminal, RT = retromargin teeth. **A, B** and **C** share the scale bar.

###### Distribution.

**Figure 9.**
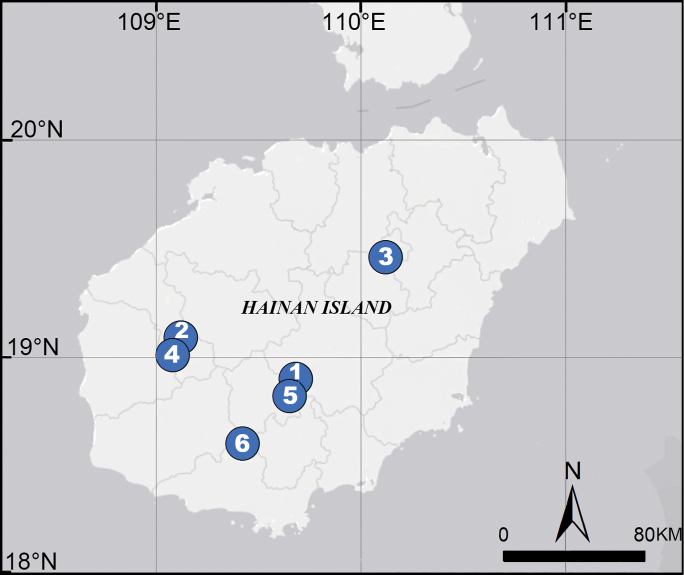
Distribution of *Flexicrurum* species in Hainan Island, China. 1. *F.wuzhishanense* Li & Li, sp. nov., 2. *F.yangjiao* Li & Li, sp. nov., 3. *F.qishi* Li & Li, sp. nov., 4. *F.minutum* Tong & Li, 2007, 5. *F.flexicrurum* Tong & Li, 2007, 6. *F.longispina* Tong & Li, 2007.

Hainan Island, China (Fig. 9).

###### Remarks.

The female was matched with the holotype male on the basis of proximity of its collection location to the type locality (only about 30 km away), similarities in somatic morphology with the holotype male, and from DNA barcoding data.

## Supplementary Material

XML Treatment for Flexicrurum

XML Treatment for Flexicrurum wuzhishanense

XML Treatment for Flexicrurum yangjiao

XML Treatment for Flexicrurum qishi

XML Treatment for Flexicrurum minutum
